# Human 3D brain organoids: steering the demolecularization of brain and neurological diseases

**DOI:** 10.1038/s41420-023-01523-w

**Published:** 2023-07-03

**Authors:** Yogita K. Adlakha

**Affiliations:** 1 Amity Institute of Molecular Medicine and Stem Cell Research, Amity University, Noida, Uttar Pradesh India; 2 Maternal and Child Health Domain, Translational Health Science and Technology Institute (THSTI), Faridabad, Haryana India

**Keywords:** Neural stem cells, Developmental neurogenesis, Molecular neuroscience

## Abstract

Understanding of human brain development, dysfunction and neurological diseases has remained limited and challenging due to inability to recapitulate human brain-specific features in animal models. Though the anatomy and physiology of the human brain has been understood in a remarkable way using post-mortem, pathological samples of human and animal models, however, modeling of human brain development and neurological diseases remains a challenge owing to distinct complexity of human brain. In this perspective, three-dimensional (3D) brain organoids have shown a beam of light. Tremendous growth in stem cell technologies has permitted the differentiation of pluripotent stem cells under 3D culture conditions into brain organoids, which recapitulate the unique features of human brain in many ways and also offer the detailed investigation of brain development, dysfunction and neurological diseases. Their translational value has also emerged and will benefit the society once the protocols for the upscaling of brain organoids are in place. Here, we summarize new advancements in methods for generation of more complex brain organoids including vascularized and mixed lineage tissue from PSCs. How synthetic biomaterials and microfluidic technology is boosting brain organoid development, has also been highlighted. We discuss the applications of brain organoids in studying preterm birth associated brain dysfunction; viral infections mediated neuroinflammation, neurodevelopmental and neurodegenerative diseases. We also highlight the translational value of brain organoids and current challenges that the field is experiencing.

## Facts

1. Brain organoids represent complex human based in vitro tools to study brain development, dysfunction and disorders.
2. Human brain organoids offer better visualization of brain structure and function than two-dimensional monolayer cultures and animal models.
3. It is possible to study interorgan interactions, preterm and infection associated neurodevelopmental impairment in extreme details with advanced methods.

## Open questions

- Can human brain organoids become translational in vitro tool also where they can be employed for production of neurotransmitters such as dopamine?
- To represent overall spatial and temporal cytoarchitecture of brain with its microenvironment, protocol advancement is still warranted.

## Introduction

Human brain is the most complex and greatly developed organ in multicellular organisms [1]. Distinct complexity of human brain contributed by genomic changes and protracted development has made it a daunting task to understand human brain development and neurological disorders using animal models [2]. Despite this, most of our knowledge about human brain has been acquired from post-mortem, pathological samples and animal models, which do not completely recapitulate the complex human brain features and initiation of disease [3, 4]. However, recent surge in stem cell and engineering technologies have provided a boost to developing human based tools to study the human brain development and disease [5, 6].

Next efforts in stem cell technology are focused on developing methods to direct the differentiation of pluripotent stem cells (PSCs) into desired brain specific cell types. Earlier, adherent two-dimensional (2D) cell culture systems were derived from PSCs for studying neuronal development. However, 2D models lack the endogenous tissue architecture, thus poses challenges to modeling of developing brain and to study of complex cell interactions in vitro [7]. Tissue architecture and intercellular interactions are highly important for understanding neurodevelopmental disorders where multiple cell types are affected [8]. In this scenario, development of new 3D culture methods for differentiation of PSCs into 3D brain organoids has permitted the investigation of normal brain development and pathogenesis of neurological diseases [9].

Brain organoids are three-dimensional multicellular structures, derived from PSCs under specific in vitro conditions where they self-organize themselves to some extent and simulate the in vivo brain regions partly [3]. In recent years, there have been tremendous boost in technological advances to generate brain organoids that resemble specific human brain regions such as neocortex, hippocampus, hypothalamus, and midbrain [10, 11] (Table 1).

**Table 1:** Different molecular cocktails to generate brain region-specific organoids.

Sr. No.	Brain region-specific organoids	Cell type used	Intrinsic signaling or extrinsic inductive signals	Reference
1	Cerebral Cortex	Mouse ESCs, hESCs and human iPSCs	Dkk-1 (Wnt inhibitor), LeftyA (NODAL inhibitor) and soluble BMPRIA-Fc	[25]
		Human H9 ES and iPSCs	Intrinsic signaling	[9]
		Human ESC	IWR1e (Wnt inhibitor) and SB431542 (TGFβ inhibitor)	[19]
		Human iPSC	Dorsomorphin (BMP inhibitor) and SB-431542, FGF2 EGF, BDNF and NT3	[26]
		Human iPSC	Recombinant mouse Noggin, FGF2, rhDkk1, EGF, BDNF, GDNF and dibutyryl-cAMP	[111]
		Human iPSC	Dorsomorphine, A83-01, WNT-3A, CHIR99021, SB-431542, ascorbic acid, BDNF, GDNF, TGFβ and cAMP	[27, 28]
		Human ES (H9 or H1) and iPSCs	Intrinsic signaling with CHIR 99021	[31]
2	Midbrain	Human ESC lines H1 and H9	CHIR99021, Noggin, SB-431542, SHH-C25II, FGF8, BDNF, GDNF, ascorbic acid, and db-cAMP	[30]
		Human iPSC	LDN-193189, SB-431542, SHH, purmorphamine, FGF-8, CHIR99021, BDNF, GDNF, ascorbic acid, TGFβ and c-AMP	[27]
		Human NESCs	CHIR-99021, purmorphamine, ascorbic acid, BDNF, GDNF, ascorbic acid, TGFβ and db c-AMP	[136]
3	Hypothalamus	Human iPSC	SB431542, LDN193189, thioglycerol, WNT3A, SHH, Purmorphamine, FGF-2 and CTNF, BDNF, GDNF, ascorbic acid and c-AMP	[27, 28]
4	Hippocampus	Human ES cells	Wnt inhibitor (IWR1e), SB431542, CHIR 99021, BMP4, chemically defined lipid concentrate	[11]
5	Anterior pituitary gland	Mouse ES cells	BMP4, SAG, DAPT, FGF10	[137]
		Human ESCs	Chemically defined lipid concentrate, monothioglycerol, BMP4, SAG, FGF2	[138]
6	Cerebellum	Human ESCs	SB431542, FGF2, SDF1 and FGF19	[139]
7	Spinal cord	Mouse ESCs	All-*trans* RA, SAG, cyclopamine	[140]
		Mouse ESCs, Human iPSC	bFGF, CHIR99021, retinoic acid, BMP4, DAPT	[141]
8	Retina	Mouse ESCs, Human ESCs	IWR1-endo (Wnt inhibitor), FBS, SAG, CHIR99021 (Wnt agonist)	[142]
		Human iPSCs	FBS, Taurine, all-*trans* RA	[143]
		Human iPSCs	FBS, Taurine, all-*trans* RA	[144]
		Human ESCs (H9), Human iPSCs	FBS, Taurine, insulin-like growth factor 1, all-*trans* RA, 9-*cis* retinal	[145]

Brain organoids can be employed to study normal and abnormal developmental processes implicated in infectious diseases, genetic disorders, and cancer. In this review, we summarize new method advances for generation of brain organoids from PSCs. We discuss the applications of brain organoids in studying preterm birth and viral infections associated brain dysfunction and neurological diseases. We also summarize the translational value and the challenges that the brain organoid field is tackling.

## Advancement in methods to generate brain organoids

**Fig. 1:**
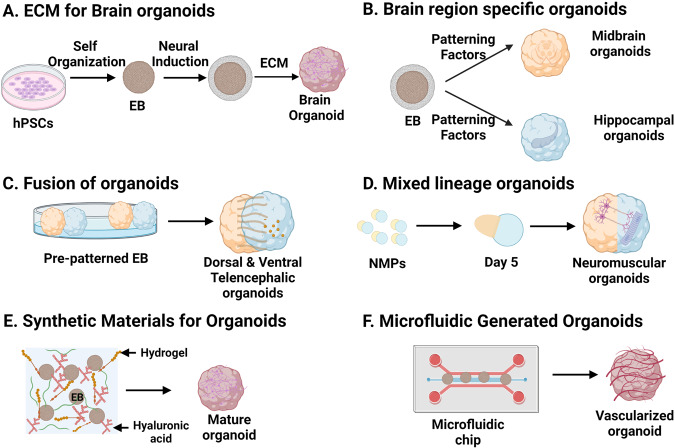
Methodological advancement in brain organoid generation. **A** Simple methods involving the minimal media and extracellular matrix generate self-organized whole brain organoids. **B** A cocktail of patterning molecules generates brain region-specific structures such as midbrain and hippocampal organoids. **C** Fusion of different organoids permits the study of interaction and migration between various cell types of different brain regions. **D** Mixed lineage organoids such as neuromuscular organoids allow the investigation of the interorgan interactions. **E** Synthetic materials enhance maturation of organoids. **F** Microfluidics develops vasculature in organoids.

**Fig. 2:**
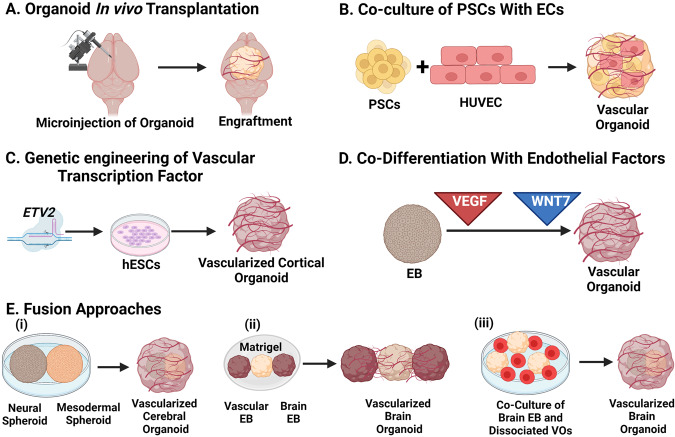
Schematic of different strategies to vascularization of brain organoid. **A** Microinjection of brain organoids in mice brain led to engraftment and invasion by host vasculature. **B** Co-culture of PSCs with endothelial cells and **C** genetic engineering of inducible transcription factor ETV2 in hESCs led to vascular system development in brain organoids. **D** EBs early fed with endothelial factors such as VEGF and later with WNT7 display vascularization along with neuronal differentiation. **E** Different fusion approaches led to brain organoid vascularization include fusion of (i) neural and mesodermal spheroid (ii) brain EB sandwiched with vascular EB in a Matrigel droplet (iii) brain EB and dissociated vascular organoids.

Initial seminal work on 2D monolayer culture differentiation [12–16] and landmark studies of Sasai and Clever’s group on 3D culture have set up the platform for the development of pioneer protocols to generate brain organoids [17, 18]. In general, brain organoids can be generated using two major techniques including self-organization of hPSCs and employing external inductive signals. Sasai and Knoblich groups have pioneered the 3D brain organoid culture systems by using neural inductive molecules and the extracellular matrix (matrigel), respectively [9, 19]. Though human brain organoids emulate the essential features of fetal brain, yet, limitations of current organoid methods including interior hypoxia and cell death have paused the derivation of 3D brain structures recapitulating the late fetal developmental stages [10]. Strikingly, Gordon et al have developed a method to derive 3D human cortical organoids, which show signals of postnatal stages [20]. We further discuss advancement of methods in culturing the brain organoids (Figs. 1 and 2).

### Extracellular matrix (ECM) for cerebral organoids

Lancaster et al, in their remarkable study, have exploited self-organizing and self-patterning properties of hPSCs which led to generation of cerebral organoids from hPSCs in presence of ECM [9]. This study was based on concept of neural default pathway that suggests the acquisition of default neuroectodermal fate by hPSCs in absence of external inductive signals [21]. However, Sasai group cultured hESCs in presence of external inductive signals such as TGFβ inhibitor and Wnt inhibitor for the initial patterning and later used matrigel at low concentration in presence of high (40%) O_2_ concentration [19].

### Extracellular inductive signals for region specific organoids

Brain is a heterogeneous tissue, which is made up of several regions. Brain development is accompanied by simultaneous formation of different brain regions, which takes place by the patterning of neural tube through the concerted play of secreted morphogens/patterning factors from the organizer region [21]. Same principle has been applied by researchers to develop in vitro methods to guide the cells in the organoids to adopt a specific brain region fate by supplementing the culture media with the extracellular inductive signals or patterning factors which will either suppress or stimulate a specific developmental signaling pathway at distinct developmental stages [2].

Robust protocols have been developed for region specific 3D brain organoids depending on the anterior–posterior (AP) and dorso–ventral (DV) polarity of the specific region [16]. Two patterning factor cocktails guide neuroectodermal identity along AP and DV axis of neural tubes. Since, Wnt and TGFβ pose negative effects on mammalian neural differentiation [22, 23], supplementation of the Wnt and Nodal antagonists (Dkk1 and LeftyA) in serum-free floating culture of embryoid body-like aggregates (SFEB) of ES cells for first 5 days results in selective neural differentiation (∼90%). However, Wnt3a treatment during the later stages favors the pallial telencephalic fate, whereas Shh augments basal telencephalic differentiation [24]. Eiraku et al. have used Wnt, Nodal antagonists and BMPRIA-Fc in their modified SFEB with quick re-aggregation (SFEBq method), and efficiently produced polarized cortical neuroepithelia from mouse, human PSCs [19, 25]. Therefore, the first patterning factor cocktail was established, aiming at coherent neural induction and AP identity along the neural tube. Several studies have displayed efficient conversion of hPSCs to neuroectodermal identity by suppressing TGF-β and BMP signaling using the dual-SMAD inhibition approach [16, 26, 27]. Dose dependent early insulin treatment and Wnt activation have been used to generate various brain regions including midbrain, thalamic, hypothalamic and cerebellar fates [27–30]. For second patterning factor cocktail, pulse of Wnt activity at later stages can give rise to telencephalic organoids [27, 28, 31]. While hippocampal tissues can be generated by Wnt and BMP treatment [11], ventral fates of telencephalon, hypothalamus and midbrain can be derived using early SHH activation [27, 30, 32–34]. Beside these inductive signals, a few other morphogens have also been included in the differentiation media. For example, rostralisation of the cortical organoids was achieved with the treatment of FGF8 [19, 25]. While, same inductive signal can pattern different brain regions in a density gradient and temporal manner, therefore, for specific brain regions, establishing a chemically-defined patterning factor cocktail and order of treatment in a low cost and reproducible manner are the recent roadblocks [2]. Despite these challenges, brain region specific organoids are helpful in elucidating the effect of genetic mutation and treatment paradigms for a brain region specific disease [30, 35, 36]. However, brain region specific organoids lack the inter-region interactions and therefore, investigation of inter-region interactions using these methods becomes challenging. Organoid fusion approach has emerged as a possible solution to study inter-region interactions, and this technique is well discussed elsewhere [2] (Fig. 1).

### Enhance the maturation of organoids

Efforts to obtain functional, mature neurons and glial cells in organoids resulted in long term culturing of organoids in 3D culture media. Long term cultivation helped in attaining calcium activity at 50 days [26, 27], spontaneous excitatory post-synaptic currents at 4 months [37] and acquisition of glial cells along with mature neurons, astrocytes, synapse, and dendritic spines at more than 6 months [38, 39]. Prolonged culturing time encourages study of mature cell types in vitro.

However, several challenges associated with prolonged culture include insufficient oxygen supply to interior part of organoids, hypoxic inner cores and cell death of the tissue. Though several approaches such as supplying increased oxygen levels in the incubator, using spinning bioreactor or gas permeable culture plates have been adopted to boost oxygen supply to the inner core, yet the maturation of healthy brain organoids were similar to mid gestation periods. Recently, Guo-Li Ming’s group has developed a method of slicing the 45-day-old neocortical organoid into sliced neocortical organoids which upon further culturing showed reduced inner hypoxia, diminished cell death, sustained neurogenesis and formation of deep and upper layer neurons over long-term cultures. The sliced organoid architecture mimics the embryonic human neocortex at third trimester of gestation [10]. Recently, Pasca and colleagues also performed long term culture of cortical spheroids and grew them up to 694 days. These spheroids displayed isoform switching in the histone deacetylase complex and NMDA receptor subunits that mark the transition from prenatal to early postnatal stages of brain development between 250 and 300 days [20].

### Mixed lineage tissue organoids

Organoids not only mimic one tissue type but also recapitulate the phenotypes of two or mixed lineage tissues such as brain and retina and thus allows the investigation of interorgan interactions in vitro. Two recent papers have developed two lineage tissue organoids. Jay Gopalakrishnan’s group has explored an innovative way to develop bilaterally symmetric optic vesicles from forebrain organoids. Their method includes culturing single cell iPSCs in neural induction medium till the formation of neurospheres, followed by culturing in neurosphere medium with addition of retinol acetate. Early supplementation of retinol acetate favored early optic vesicle development in brain organoids [40]. Mina Gouti and colleagues in their seminal study used hPSC lines to generate neuromesodermal progenitors and allowed them to form 3D aggregates in neurobasal medium with several growth factors. The resultant neuromuscular organoids (NMOs) form functional neuromuscular junctions among skeletal muscle cells, spinal cord neurons, and Schwann cells simultaneously (Fig. 1) [41]. Another study differentiated iPSC lines into neuromesodermal progenitors and derived sensorimotor organoids from them. These organoids contained cells of neuronal origin, such as sensory and motor neurons, astrocytes and of mesodermal origin such as microglia, endothelial cells and skeletal muscle. They also reported physiologically active neuromuscular junctions between the motor neurons and skeletal muscle [42].

### Specialized and vascularized brain organoids

Human brain organoids generated using methods discussed so far, lack vasculature; this results in restricted supply of oxygen and nutrients to the inner part of the organoids that lead to necrosis and cell death of the inner part of organoids, limiting the organoid growth [43, 44]. Absence of vasculature also impacts signaling, differentiation and maturation of brain progenitor cells [45]. Therefore, developing vasculature in organoids is important to achieve diverse, differentiated and reproducible cell repertoire (Fig. 2) [46, 47].

In this direction, first efforts were shown by Fred Gage group by transplanting human brain organoids into adult mouse brain, which provides in vivo physiological conditions to organoids and led to improved neuronal differentiation and maturation, glial cells generation, functional synapse, and blood vasculature in the grafts [48]. Next approach involves engineering of hESCs to transiently overexpress human ETS variant 2 (ETV2), leading to the emergence of ETV2-expressing cells in human cortical organoids, and finally displayed the formation of a complex vascular-like network in cortical organoids. Vasculature acquired organoids displayed numerous characteristics of blood-brain barrier (BBB) [49]. In another approach, PSCs were co-cultured with human umbilical vein endothelial cells (HUVEC) to form cortical organoid. These organoids also displayed the formation of tubular vascular system [50]. Notably, in both studies, vascular organoids exhibit functional vasculature, decreased cell death, improved maturation and synaptic connections after transplantation in mice [49, 50]. In addition, EBs cultured in VEGF rich media led to co-differentiation of vascular endothelial cells (ECs) along with neuronal cells in cerebral organoids. Addition of VEGF and WNT7a led to formation of segregated blood vessel-like structures in cerebral organoids with BBB characteristics [51]. Another alternative involves propagating neural and vascular cell types separately as spheroids followed by their fusion. Researchers have fused either neural and mesodermal spheres [52] or vascular and brain organoids [53, 54] and cultivated them for long time. Though the blood vessel formation and maturation was successful after fusion, yet blood vessel network was least responsive to pro- and anti-angiogenic conditions [52]. To avoid regional localization of blood vessels in brain organoids, Ahn et al. dissociated blood vessel organoids and co-cultured with brain organoids using neural induction media. This led to formation of long blood vessels with pericytes; however, astrocytes were not closely associated with blood vessels [55]. Beside these approaches, microfluidics technology, ECM proteins and synthetic materials have been employed to enhance the vascularization of cerebral organoids [31, 56, 57].

### Functional materials in organoid induction

It has been a great challenge to recapitulate the brain ECM in 3D cultures, which led to immense usage of natural materials such as Matrigel [58]. However, its poorly defined composition, xenogenic nature, batch to batch variation and lack of reproducibility has permitted researchers to explore alternatives of matrigel [59]. Recent advances in synthetic materials have led to the development of better ECM-like materials, which are chemically defined, greatly modifiable, reproducible and xenogenic-free alternatives [59, 60]. For instance, decellularized adult porcine brain ECM has been used to grow ESC-derived brain organoids, which exhibit mature morphology on day 40 [61]. Similarly, collagen hydrogel also improves maturation of neuronal network in 3D neuronal organoids, which co-developed excitatory, inhibitory neurons and glia cells [62]. In the next leap, a composite of recombinant spider silk with human laminin 111 reduces cellular stress and intra-organoid variability and enhances neuronal maturation in cerebral organoids [63].

Researchers have employed polyethylene glycol (PEG) and generated homogeneous 3D neural tube constructs [64]. Strikingly, without addition of any neural inductive signals, Cell-Mate3D hydrogels (a mixture of hyaluronic acid (HA) and chitosan) have generated cerebral organoids in just 10-14 days [65]. In addition, HA, an important component of brain ECM, in combination with heparin causes caudalization of iPSC derived brain organoids [66]. Besides these materials, alginate either alone or in combination with HA also stimulates neural fates in 3D culture of PSCs [67]. The synthetic materials can also deliver various growth factors important for regulation of vascularization and microenvironment such as VEGF, which modulates the brain organoid development and promotes long term survival [68]. These advances in synthetic materials to mimic brain ECM help in the development of improved brain organoid models.

### Microfluidics in organoid induction

Microfluidics refers to a miniaturized microfluidic cell culture device that enables organ-on-a-chip system by feeding live cells in continuously perfused chambers [69, 70]. This technology offers to control the tissue’s physicochemical microenvironment and mimics cytoarchitecture and vascular perfusion of the tissue to a great extent [56, 58]. The application of microfluidic technology in vascularization of organoids is immense [71–73]. Recently, a combination of microfluidic device polydimethylsiloxane (PDMS) chip and human brain ECM has resulted in improved neurogenesis and better electrophysiological function in 3D brain organoids [74]. The reason for improved neurogenesis could be reduction of stress as seen by Seiler et al. They observed reduced glycolytic and endoplasmic reticulum stress in cortical organoids grown on automated, PDMS fabricated microfluidic cell culture platform [75]. The microfluidic technology can also be used for co-development of cells. For instance, co-development of hPSC derived pericytes, endothelial cells and cerebral organoids in 3D printed microfluidic platform leads to the formation of organized vascular networks, which interacts with cerebral organoids and perfuse them [57]. Not only co-development but brain region specific organoids have also been generated using microfluidic chips. For instance, human iPSCs enveloped in microcapsules, generated brain region-specific organoids, which were assembled into brain assembloids using multi-layered microfluidic chip with dynamic fluid flow. These assembloids were composed of cortical, hippocampal, and thalamic organoids, which recapitulated the neural migration and interaction [76]. In addition, perfusion of brain organoids and delivery of oxygen and nutrients can be improved by maintaining active flow of media in the microfluidic chips using syringe pump, peristaltic pump and acoustofluidic minibioreactor [77–80]. Microfluidics grown brain organoids have recently been employed in toxicity studies. For instance, cannabis exposure of brain organoids on one-stop microfluidic chips led to decreased neuronal maturation, diminished neurite outgrowth and reduced spontaneous firing in organoids [81]. Micro fabricated petri dish compartment coated with PDMS was used to model the folding of human brain organoids, which displayed folds in the first week of development [82]. Thus, microfluidics combines the fields of fluid dynamics, synthetic polymer chemistry with biology to study brain structure, physiology, development and drug discovery.

## Applications of brain organoids

### To study neurodevelopmental impairment associated with preterm birth

**Fig. 3:**
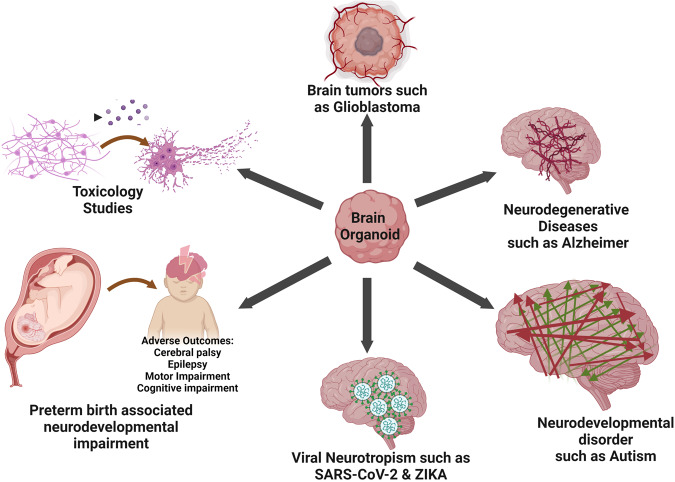
Applications of brain organoids. Brain organoids can be used to study preterm birth-associated neurodevelopmental impairment, viral neurotropism such as SARS-CoV-2 and Zika, Neurodevelopmental and neurodegenerative disorders, brain tumors such as glioblastoma and for toxicology studies.

Preterm birth is referred to as birth before 37 weeks of gestation period [83]. This contributes to the ~15% neonatal deaths and thus represents the single leading cause of neonatal deaths worldwide [84]. Out of survivors, ~80% very early preterm infants (birth before 28 weeks of gestation) confront varied degree of neurodevelopmental impairment into later life. After birth, preterm infant can encounter hypoxia, which can develop an acute ailment called encephalopathy of prematurity [85] that lead to impairment of complex behavioral and cognitive functions (Fig. 3) [86].

Brain organoids permit the modeling of these complex functions and umbilical cord tissue and cord blood can be used to generate iPSC derived brain organoids [87]. The molecular mechanisms underlying hypoxia mediated neurodevelopmental impairments in very early preterm infants are not understood well. Recently, Pasca et al have delineated the effect of oxygen deprivation on corticogenesis using human brain-region-specific organoids. Oxygen deprivation reduces the population of cortical intermediate progenitors, which contribute immensely to the human cerebral cortex expansion. Notably, this reduction was associated with the unfolded protein response (UPR) pathway as addition of a small-molecule inhibitor of the UPR pathway rescues the decrease in intermediate progenitors following hypoxia [85].

### To study viral infection mechanism/neurotropism

Infectious diseases are caused by pathogens, which regularly emerge and spread with pandemic or epidemic potential and shatter human life. The recent outbreak by novel human coronavirus SARS-CoV-2 from China and Zika virus in America has emerged as pandemic and epidemic, respectively. They devastated human species by causing COVID-19 and microcephaly, respectively.

Detection of SARS-CoV-2 and ZIKV RNA in some patient’s brains and a suspected association between the ZIKV outbreak and congenital microcephaly in newborns has shown the neurotropism and neurotoxic effects of these viruses. There is no direct experimental evidence, which concludes the neurotoxic and birth defects of SARS-CoV-2 and ZIKV, respectively and therefore, has led to employing 3D brain models to reveal viral infection mechanisms [88, 89]. Recently, a few studies have validated that SARS-CoV-2 infects cortical neurons in brain organoids, iPSC-derived hNPCs, neurospheres, brain spheroids, astrocytes and microglia [90–94] and caused metabolic alterations, microglia mediated synapse engulfment and neuronal cell death [58, 60]. Contradictory, other studies suggest the sparse or rare infection of neurons, astrocytes and microglia by SARS-CoV-2 [95, 96]. However, choroid plexus organoids show robust infection of SARS-CoV-2 followed by cell death and compromised barrier integrity. Abundance of SARS-CoV-2 entry receptors in choroid plexus cells may lead to the dramatic infection of choroid plexus organoids [96]. Similarly, cerebral organoids have also advanced our understanding of infection mechanisms of ZIKV. ZIKV efficiently replicates in brain organoids and diminishes their growth [97]. ZIKV selectively infects NPCs and forebrain organoids and causes reduced proliferation of NPCs followed by enormous cell death, reduced neuronal volume and organoid size [27, 66, 67]. In organoids, ZIKV activates innate immune receptor TLR3 and depletes NPCs [98]. Mechanistically, in forebrain organoids, NS2A protein of ZIKV diminishes proliferation of radial glial cells and leads to adherens junction (AJ) complex deficits [99]. Recently, ZIKV infection in brain organoids led to accumulation of Aβ plaques, increased p-Tau expression and expedites AD pathology [100].

Further, the disparity between different studies about the cell type specific infection of these viruses could be because of usage of different viral strains such as wild type, spike-pseudotypisized viral vector, delta and omicron isolates [93, 101, 102]. Besides these mechanistic studies, brain organoids have also been employed to reveal strain specific neurotropism of ZIKV and for therapeutic development [103, 104]. For instance, SARS-CoV-2 infection in brain organoids can be prevented by halting ACE2 receptor with antibodies or by culturing organoids with cerebrospinal fluid from a COVID-19 patient [105]. Recently, Sofosbuvir, a FDA approved antiviral (anti-hepatitis C) prevented SARS-CoV-2 replication, decreased neuronal death and rescued synaptic integrity in infected cortical organoids [92]. Notably, Sofosbuvir also protected human NPCs and 3D neurospheres from ZIKV mediated cell death [106]. Strikingly, brain organoid defects elicited by ZIKV were rescued by the type I interferons and RNAi enhancer enoxacin [97, 107]. Together, these studies have highlighted the use of human brain organoid models in revealing neurotropism and delineating the molecular mechanisms underlying viral infections of brain, and also in screening potential therapeutic candidates.

### To understand mechanisms underlying neurological diseases

Neurological diseases include neurodevelopmental and neurodegenerative diseases and mechanistic understanding and drug development of neurological diseases has hampered due to inaccessibility of patient brain tissues at disease initiation stage. This has led to development of 3D brain organoids, which can recapitulate the complexity, physiology and pathology of the human brain. We describe this section using specific examples of neurodevelopmental and neurodegenerative disorders (Fig. 3), while most of these diseases have been mentioned in Table 2.

**Table 2:** Disease modeling using 3D human brain organoids and challenges.

Neurological disease	Challenge	Brain region-specific organoids	Growth factors/ECM used	Cellular phenotype/phenomenon	Reference
Microcephaly-related disease conditions					
Microcephaly	Mouse models do not mimic the severity of Microcephaly because of smaller brain and limited expansion	Cerebral organoids	Y-27632, N2 supplement, Heparin, Matrigel, B27 supplement without vitamin A, Insulin	Progenitor cells with decreased radial glial cells show premature neural differentiation	[9]
	Microcephaly patients with Aspm mutations possess extremely reduced brain size, which is quite difficult to mimic in animal models	Cortical organoids	endo-IWR1, LDN-193189, SB431542, heparin, BDNF, GDNF, cAMP, matrigel	Patient-derived cortical organoids displayed proliferation deficient progenitors, less mature neurons and abnormal cortical lamination, neuronal dysfunction	[37]
Lissencephaly (Miller Dieker Syndrome)	Absence of outer radial glial cells in developing rodent cortex leads to milder phenotypes in Pafah1b1+/− mice as compared to human patients	Cerebral organoids	Y-27632, WNT inhibitor, TGF-β inhibitor, FBS, Matrigel and heparin	Defective cell migration, massive apoptosis of founder neuroepithelial stem cells, Defective mitosis in outer radial glia	[146]
Neurodevelopmental disorders					
Bipolar disorder	Mechanistic understanding of the Bipolar Disorder is unclear due to the scarcity of causal genes displaying robust effect sizes	Cerebral organoids with dorsal forebrain identity	STEMdiff Cerebral Organoid Kit & maturation kit with BDNF	Cerebral organoids derived from diseased and control iPSCs were not apparently different. Bipolar cerebral organoids show precise defects in response to stimulation and depolarization	[147]
Bipolar disorder/schizophrenia	Underlying cellular mechanisms for the pathogenesis of Bipolar disorder are not clear due to heterogeneity and technical constraints on cellular complexity of the brain.	Cerebral organoids	SB431542, LDN-193189, Y-27632, Matrigel	Accelerated neuronal differentiation, increased GABAergic specification, decreased cell proliferation following reduced Wnt signaling	[148]
Developmental and epileptic encephalopathies (DEE)	DEE is a heterogeneous disorder associated with intractable seizures, abnormal brain development, and functional abnormalities. Every patient has distinct genetic background and brain development, which cannot be modeled in a rodent model	Cerebral organoids	bFGF, Y-27632, Matrigel, Insulin, CHIR-99021, vitamin C	Defective DNA damage response, activation of Wnt pathway, abnormal cortical differentiation with disproportionate glutamatergic and GABAergic neurons and increased astrogenesis	[149]
Timothy syndrome	In vitro modeling of migration of interneurons and their functional integration into human cortex during fetal development remains a challenge	Forebrain spheroids containing cortical (hCS) and subpallium spheroids (hSS)	hCS: dorsomorphin, SB-431542, FGF2, EGF, BDNF, NT-3 hSS: FGF2, EGF, IWP-2, SAG, NT-3, allopregnanolone, retinoic acid, BDNF	Increased saltation frequency led to defective migration of interneurons	[32]
Tuberous sclerosis complex (TSC)	Highly heterogeneous developmental disorder, cortical tubers are not formed in rodent models	Cortical spheroid	Dorsomorphin, SB431542, EGF, FGF, BDNF, NT-3	Hypertrophic NPCs, enlarged and dysmorphic neuronal cells with enhanced glia production	[150]
Angelman syndrome (AS)	AS mice models show synaptic dysfunction and impaired plasticity; however, mechanisms in AS patients remain unclear	Cortical organoids	Dorsomorphin, SB431542, B27 without vitamin A, EGF, FGF-basic, BDNF, GDNF, NT3, db-cAMP	Hyper-excitability and synchronous firing, UBE3A via degradation of calcium- and voltage-dependent big potassium (BK) channels inhibits neuronal hyper-excitability	[151]
Rett Syndrome	Non-availability of embryonic or perinatal post-mortem tissues from Rett Syndrome patients	Cerebral organoids	Y-27632, SB431542, dorsomorphin, Matrigel, B27 without vitamin A, B27 with vitamin A	increased ventricular area, reduced radial thickness and massive increase of neural progenitor cells followed by abnormal migration of neurons and aberrant neurogenesis	[152]
	Lack of studies on influence of MeCP2 mutation on human interneuron development and function	Human medial ganglionic eminence (MGE) and cortical organoids	LDN-193189, SB-431542, XAV-939, Y-27632, hMGEO: B27 supplement without vitamin A, recombinant SHH, purmorphamine hCO: B27 supplement with vitamin A, BDNF, ascorbic acid, cAMP	BET inhibitor, JQ1, improves the functional deficits of RTT interneurons in brain organoids by showing area-scale synchronization of calcium surges and rescuing the dysregulated transcriptome	[153]
	RTT phenotypes are widespread and various kind of mutations present the disease phenotypes differently	Dorsal and ventral forebrain organoids	Dorsal Forebrain: Dorsomorphine, A83-01, CHIR99021, SB-431542, Heparin Ventral: SB-431542, LDN-193189, IWP2, SAG and Heparin. BDNF, GDNF, dibutyryl cAMP and ascorbic acid	Premature development of the deep-cortical layer with less proliferating neural progenitor/proliferative cells along with the impairments of interneuron’s migration, dysfunctional RTT neurons, defects in production of medial ganglionic eminence (MGE) progenitors in ventral organoids	[154]
	Genetic mutation of MeCp2 gene impairs neurodevelopment, still therapeutic treatment for this syndrome is not available	Cortical organoids	SB431542, Dorsomorphin, Y-27632, FGF2, EGF, BDNF, GDNF, NT-3, ascorbic acid and dibutyryl-cAMP	In MECP2‐KO cortical organoids, two lead compounds rescued synaptic pathways and neural network function	[155]
Down syndrome	Technically inaccessible pathological samples, presence of asynchronous and heterogeneous disease phenotypes in in vitro culture	Cerebral organoids	N2 supplement, Matrigel	Diminished neurogenesis, reduced proliferation and decreased expression of cortical neuronal markers in layer II and IV in the subcortical regions	[156]
	Vague knowledge of role of Olig genes in GABAergic neuron generation and inconsistencies in recapitulating DS-related genotype–phenotype relationships and contradictory results from human samples	Ventral forebrain organoid	SB431542, Noggin, Matrigel, Laminin, B27-RA, FGF2, hLIF, CHIR99021, Y-27632, SHH, purmorphamine, BDNF, GDNF, dibutyryl-cyclic AMP and ascorbic acid	Overproduction of OLIG2+ Progenitors, disproportionate interneuron production, recognition memory disrupted in neuronal chimeric mice	[157]
ASD	Phenotypic heterogeneity and absence of behavioral phenotypes of ASD in rodent models	Telencephalic organoids	B27 supplement without vitamin A, N2 supplement, 2-mercaptoethanol, Y-27632, FGF2, Noggin, rhDkk1, EGF, ascorbic acid, BDNF, GDNF and dibutyryl-cAMP	Elevated neuronal maturation and synaptic overgrowth, Massive inhibitory synapses in ASD-derived neurons, balance between the number of excitatory and inhibitory neurons in ASD-organoids was disturbed	[111]
	Heterogeneous population of neurons expressing forebrain, midbrain, and hindbrain markers was present in monolayer neuronal culture system	Cerebral organoid	DKK-1, BMPRIA-Fc, SB431542, N2 supplement, laminin, and fibronectin, B27 and l-glutamine	Radial glia progenitor cells, GABAergic and glutamatergic neurons were present in cerebral organoids	[114]
	To integrate the finding that DNA methylation patterns of GAD1 are dysregulated during development from patient’s post-mortem brain and rodent models	Cerebral organoids	Y-27632, Matrigel	Early development follows a diverse methylation patterns in GAD1 in ASD cerebral organoids	[158]
	Lack of understanding of cell lineages and molecular pathways implicated in telencephalic development in ASD	single neural rosettes derived telencephalic organoids	N2 Supplement, Heparin, B27 Supplement with vitamin A, Dorsomorphin, SB431542, EGF, FGF, BDNF, GDNF and NT-3	Smaller size of SHANK3-/-organoids, decreased population of neurons with smaller nuclei sizes, decreased number of excitatory synaptic puncta	[159]
ASD	Mechanisms underlying valproic acid contribution to accelerating ASD risk in human are not known	Forebrain organoids	SB431542, LDN193189, Y27632, Heparin, CHIR99021, WNT-3A, Matrigel, insulin, Forskolin, Ascorbic Acid, BDNF, GDNF	Disturbed synaptic transmission in VPA treated organoids, differentially expressed genes enriched in calcium, and potassium signaling pathways, oxytocin signaling, synaptic transmission, and neural development	[160]
	How interactions among gene–environment increases ASD risk are unknown	Brain organoids	FGF, EGF, GDNF, BDNF	Exposure of CHD8 +/− organoids with organophosphate pesticide resulted in disturbed Neurite outgrowth and imbalance of excitatory/inhibitory neurotransmitters	[161]
Neurodegenerative diseases					
Alzheimer disease	Two-dimensional in vitro models poorly demonstrate the aggregation of extracellular protein involved in Alzheimer disease	Neural organoids	Y-27632, SB431532, IWRe1, Dorsomorphin, heparin Matrigel, B27 supplement	Amyloid beta, tau pathology and endosome abnormality is mimicked in fAD organoids, which were responsive to drug treatment	[162]
	Investigation of AD was hindered in absence of non-invasive method for harvesting brain tissue from living patients	Cerebral organoids	Y27632, Matrigel	Aβ deposits and Tau phosphorylation was observed in cerebral organoids	[163]
	AD organoids should mimic the genetic background of patients and the functional features of AD brain	Cerebral organoid	Y-27632, Matrigel	AD organoids displayed a small size, increased Aβ42/Aβ40 ratio, disrupted calcium homeostasis, and enhanced neuronal activity	[164]
	5-Hydroxymethylcytosine contribution to AD pathology has not been determined	Forebrain organoids	FGF, Matrigel, Dorsomorphin, A-83, Heparin CHIR99021,SB-431542, B27, GDNF, BDNF, TFGβ, cAMP	Forebrain organoids mimicked cellular and molecular phenotypes of AD brain. More 5hmC peaks in EBs compared to mature organoids and modulated in intragenic regions	[165]
Parkinson	2D models do not mimic the neuron- glia interaction in a spatially organized cellular architecture	Midbrain-like organoids	SB-431542, LDN-193189, CHIR99021, SAG, Y-27632, ascorbic acid, Matrigel	Midbrain organoids comprised of dopaminergic neurons, which secrete dopamine. In patient organoids, number and complexity of mDANs was decreased	[36]
	Immaturity and heterogeneity of Midbrain organoids (MO) architecture and less efficient protocol	Midbrain organoids	CHIR99021, Noggin, SB431542, Dorsomorphin, A83-01, LDN, FGF8, SAG, Matrigel	MOs comprised of homogenous distribution of dopaminergic neurons, astrocytes and oligodendrocytes	[126]
	Mutation in DNAJC6 is associated with early-onset Parkinson’s disease, but role of DNAJC6 in PD pathogenesis was unknown	Midbrain-Like organoids (MLOs)	B27, SB431542, Noggin,CHIR99021, Y27632, purmophamine, SHH-C25II, FGF8, BDNF, GDNF, Matrigel, ascorbic acid	MLOs display death of dopamine neuron, aggregation of α-synuclein, dysfunction of mitochondria and lysosomes	[166]
Frontotemporal dementia	Role of p25/Cdk5 in tauopathy using organoid model was not known	Cerebral organoids	SB431532, IWRe1,Dorsomorphin, heparin, FBS, matrigel	Tau phosphorylation was reduced and expression of synaptophysin increased in cerebral organoids derived isogenic iPSC lines	[167]
Zika virus	Inefficient organoid differentiation protocols lead to enormous variability in brain organoids	Cortical organoids and ventral telencephalic organoids	Y-27632, 40% O2, B27 without vitamin A, Growth Factor Reduced Matrigel, heparin, Leukemia Inhibitory Factor	Functional neurons display network-like activities in organoids, Increased progenitor apoptosis leads to smaller organoids size, efficacy of different drug tested	[168]
Schizophrenia	Functional role of interaction between DISC1 and Ndel1/Nde1 in brain development is difficult to delineate	Forebrain organoids	Dorsomorphine, A83-01, Matrigel, Insulin, WNT-3A, CHIR99021, SB-431542	Cell-cycle defects in radial glial cells in forebrain organoids resulted in reduced proliferation of neural stem cells in the ventricular zone and disturbed neurogenesis	[169]
Prenatal hypoxic injury	Technical challenge in accessing human fetal brain tissues and inappropriate animal models have led to poor understanding of effects of hypoxia on progenitor steady state and developmental progression during early human brain development	Cerebral organoids	Matrigel, N2 supplement, B27 supplement without vitamin A, insulin	Immense apoptosis in cerebral organoids followed by greater loss of outer radial glia progenitors and differentiating neuroblasts/immature neurons. During hypoxia, NSCs shifted to symmetric division and replete their population	[170]
Tuberous sclerosis/periventricular heterotopia	Partial recapitulation of human cortex features by mouse models have led to non-suitability of these models for understanding the mechanisms of neuronal heterotopia	Cerebral spheroids	Y-27632, N2 supplement, Heparin, Matrigel, B27 supplement without vitamin A, Insulin	Defective morphology of neural progenitor cells and altered neuronal migration in a subset of neurons	[171]
Retinitis pigmentosa		Retinal organoids	Matrigel, G418 and/or Ataluren (PTC124)	Massive cell death of rod photoreceptor at day 150 followed by thinning of the outer nuclear layer by day 180 of culture in patient-derived organoids	[172]
Brain cancers					
Brain tumor including glioblastoma and central nervous system primitive neuroectodermal tumor	Modeling of brain tumors is limited owing to genetic heterogeneity and non-suitability of animal models	Neoplastic cerebral organoid	bFGF, Y-27632, Matrigel, vitamin A	By using gene editing techniques and generating gain or loss of function phenotypes for several genes, tumorigenesis was established in cerebral organoids	[173]
Glioblastoma	Inability to recapitulate the cellular and mutational diversity of parental tumors in in vitro models and longer development time of these models.	Glioblastoma organoids (GBOs)	Patient Glioblastoma Tissue, N2 supplement, B27 supplement w/o vitamin A, insulin	Cellular composition of parental tumors recapitulated in GBOs as they display enormous heterogeneity in cell identity and morphology	[95]
	Limited donor availability for patient-derived xenograft (PDX) models and findings from mouse genetic models are not translatable to human clinical trials	Cerebral organoids	Y-27632, Heparin, N2 supplement, 1X B27 supplement w/o vitamin A, insulin, Matrigel	Invasive phenotype of genetically modified cells inside the organoid caused massive destruction of surrounding organoid structures, and display tumor pathology upon transplantation in mice	[174]
	Limited GBM models due to the lack of a normal human microenvironment and the inability to recapitulate the GBM biology	Cerebral organoid glioma	Y-27632, Heparin, N2 supplement, 1X B27 supplement w/o vitamin A, insulin, Matrigel	regional proliferation followed by massive invasion accompanied by formation of tumor microtubes and phenocopy patient glioblastoma	[175]

## Autism spectrum disorder (ASD)

ASD is a disorder of abnormal brain development, which results in language impairment, diminished social interaction and stereotypic behaviors in children [108, 109]. Immense genetic and phenotypic heterogeneity pose challenges to study ASD in animal and cellular models. However, cerebral and forebrain organoids derived from ASD patients display the clinical phenotype of ASD by showing an enlarged cortical plate thickness [110]. Telencephalic organoids derived from patient iPSCs demonstrate an enhanced cell cycle, increased expression of FOXG1 gene, which causes exaggerated production of GABAergic inhibitory neurons, highlighting an uneven GABA/glutamate neuronal population in patients [111]. Recently, cortical organoids derived from ASD patients harboring mutations in three risk genes SUV420H1, ARID1B and CHD8 also display asynchronous development of GABAergic neurons and excitatory projection neurons [112]. Dysregulated proliferation and alternative splicing causes enhanced production of inhibitory and delayed generation of excitatory neurons [113]. Moreover, telencephalic organoids generated from fetal iPSCs revealed the enrichment of ASD de novo mutations in functional enhancers, which disturb the binding sites of homeodomain transcription factors such as NR4A2, Hes1, NFIX, and Sox3 [43]. In addition, patient brain organoids also display activation of different gene networks and pathways. For instance, telencephalic organoids derived from CHD8 (autism candidate gene) heterozygous KO iPSC lines highlight the Wnt/β-catenin signaling and axonal guidance as the top significant affected pathways [114]. Patient forebrain organoids harboring a homozygous mutation in CNTNAP2 gene, display an enhanced organoid volume caused by an increased proliferation of neural progenitor cells. CRISPR-Cas9 mediated repair of CNTNAP2 mutation in organoids led to reversal of cortical overgrowth phenotypes and partially rescued transcriptional profiles [115]. Patient cortical organoids harboring 16p11.2 copy number variation (CNV) display altered ratio of neurons to neural progenitors with depletion of neural progenitors in case of deletion [76]. Transcriptomic and proteomic profiling of these organoids revealed neuron migration, actin cytoskeleton and Wnt signaling as dysregulated pathways. RhoA inhibition in cortical organoids rescued neuron migration defects but not the neurite length [116]. In addition, Orgo-Seq of patient cerebral organoids identified immature neurons and intermediate progenitor cells as crucial cells for 16p11.2 deletions [117].

## Alzheimer’s disease (AD)

AD is the primary neurodegenerative disease, associated with cognitive decline and behavioral deterioration resulting into dementia [118]. Early onset, familial AD was first modeled in cerebral organoids by mutating the APP and PSEN1 genes in the human neural progenitor cells and differentiating them in 3D cultures. These organoids display robust extracellular deposition of amyloid-β peptides (Aβ) and neurofibrillary tangles (NFT) [119]. Since then, patient cerebral organoids have been generated harboring mutations in PSEN1, APOE4 genes and recapitulating pathogenic features of AD [120, 121]. Transcriptomic analysis of patient cerebral organoids elucidates the upregulation of stress granules and dysregulated RNA metabolism [121].

Several new methods have also been developed to model the late onset AD. Recently, BBB leakage in AD was mimicked in a remarkable way by exposing the brain organoids to human serum. In this condition, key hallmarks of AD such as enhanced levels of Aβ peptides, p-tau, and synaptic loss were observed in the brain organoids [122]. In addition, herpes simplex virus (HSV-1) infection of cerebral organoids resulted in the onset of AD features without the addition of any exogenous modulators of AD [123]. Co-culture of neuron, astrocytes and microglia in a 3D microfluidic chamber recapitulated AD features and specifically microglia causes neuronal damage by secreting TNF-α and nitric oxide via IFN-γ and TLR4 mediated mechanisms [124].

## Parkinson’s disease (PD)

PD is another most common neurodegenerative disorder, which exhibits tremors, drooping posture, muscle rigidity, walking difficulty and cognitive decline in aging population [125].

Recently, Kwak et al. have developed midbrain-like organoids, which contain the dopaminergic neurons, astrocytes and oligodendrocytes and display great homogeneity and maturity. In addition, astrocytes present in the organoids break down 1-methyl-4-phenyl-1,2,3,6-tetrahydropyridine (MPTP), a representative dopaminergic neurotoxin, therefore, facilitates in vitro modeling of PD [126]. Several other conditions were also standardized for midbrain organoid generation such as the cell number, the timing of the embedding and the maturation protocol [27, 36, 127, 128]. Midbrain-like organoids generated from PD patients harboring the LRRK2-G2019S mutation revealed the increased expression of FOXA2, a transcription factor necessary for production of dopaminergic neurons, thus indicate a neurodevelopmental defect in midbrain dopaminergic neurons [36]. Isogenic midbrain organoids generated with a LRRK2-G2019S mutation also mimicked the hallmarks of PD. Gene expression profiling of 3D organoids harboring LRRK2-G2019S mutation revealed thiol-oxidoreductase (TXNIP) as one of the upregulated genes and inhibition of TXNIP rescued PD pathology in organoids [35].

Therefore, these results suggest that brain organoids not only reveal causes and pathological mechanisms of neurological diseases, but can also be employed for drug screening and to elucidate the effect of genetic, epigenetic and environmental stressors.

## Translational value for therapeutic platform

Regionally patterned brain organoids can be helpful in secreting neurotransmitters such as dopamine, acetylcholine, GABA, whose production goes awry in neurodegenerative diseases. For this overwhelming approach, one needs the well-established globally standardized protocols for organoid establishment and quality control. If these protocols demonstrate enormous robustness, reproducibility, scaling up, and turns into cheaper technology than bacteria, then the day is not far from establishing the organoid technology for cheaper production of neurotransmitters. This will have immense application for Parkinson’s patients who will be able to receive dopamine secreted by a human system instead of bacterial system.

## Current challenges

Brain organoids offer unprecedented opportunity to study development, evolution and neurological diseases; however there are a number of challenges to be overcome. One of the major limitation includes reproducibility with inter batch variations [129]. The batch effect is prominent in un-guided organoids due to variations in morphology, differentiation efficiency, and cellular compositions [38, 130]. In addition, use of different PSC lines results in variable organoid size, morphology and also compromises the reproducibility of brain organoids. Therefore, appropriate stringent controls with comparable age, genetic background and gender are essential while comparing the size of the patients derived organoids to reveal the pathological phenotype of the disease. Another limitation of the current protocols includes the limited supply of nutrients and oxygen to the core of the organoid, followed by necrosis, restricted maturation and even absence of cell types from organoids. To overcome this limitation, though several efforts have developed vasculature in organoids, yet protocols need to be refined [49, 131]. Current protocols are also unable to generate full six-layered laminar cytoarchitecture of neocortex and fully mature glial cells in organoids.

## Future perspective and conclusion

3D brain organoid field has held immense potential in bringing the in vivo tissue architecture to a dish and thus has presented innumerable opportunities for disease modeling, transplantation, drug discovery and toxicology [129]. 3D brain organoids show outstanding possibilities in comparison to 2D neural rosettes in harboring organized tissue architecture by displaying unique layers of neural progenitors and appropriate migration of differentiating neurons. Multidisciplinary approaches have led to the introduction of bioreactors, microfluidic and robotic devices, 3D printers to boost the drug screening and toxicology studies in brain organoids [79, 132]. Several protocols have emerged that allow the derivation of brain region specific organoids which mimic the specific region of the brain and thus permit the study of biological mechanisms underlying brain development and dysfunction in a spatial and temporal manner. However, protocol refinement is still needed that allows the generation of all six layers of cortical neurons in cortical organoids and all the regions of hippocampus in hippocampal organoids [133]. In addition, engineered bio-scaffolds can further help in improving the spatio-temporal migration of neurons in organoid cortical plate [134]. Heterogeneity and variability among different samples and within the same protocol is yet a robust challenge. While efforts have been initiated to tackle this challenge, it warrants future investigations on exploring exogenous patterning molecules, mini-bioreactors and mini-scaffolds to enhance homogeneity [19, 26, 27, 31, 44].

Another major area in the organoid field that requires much attention is the limited maturation of the organoids due to inefficient supply of nutrients and oxygen to the core of the organoids, which has restricted organoids to model the late fetal stages. To achieve this goal, approaches must be established that can help in developing vasculature and mature non-neuronal cell types inside the organoids. Though several approaches have been adopted including co-culture of endothelial cells, microfluidic perfusion, ectopic expression of ETV2 in brain organoids and transplanting brain organoids in rodents to accomplish in vivo vascularization, bioengineered substances such as hydrogels and nanoparticles [48, 49, 132, 135], yet, multidisciplinary integrated approaches are warranted to achieve the advanced maturation and survival of organoids. Generation of non-neuronal cell types such as astrocytes, oligodendrocytes and microglia in brain organoids further help in studying the neuron-glia interaction. Long-term culturing promotes the maturation of neurons along with the differentiation of glial cells in the organoids [20]. In addition, the field requires novel strategies to model the key hallmarks of aging to study age related neurodegenerative diseases.

In brief, integration of bioengineering, microfluidics and automation permit further technological advancement in 3D brain organoid field, which will unravel novel disease mechanisms and help in diagnostic and therapeutic development of neurological diseases.
